# A Sanitary Record of House Conditions

**Published:** 1899-09-09

**Authors:** 


### A SANITARY RECORD OF HOUSE CONDITIONS.

DR. ALLEN,[1] medical officer for the Strand, has put in operation a system of registration of the health conditions of each house within his district which seems likely to prove of the greatest service in tracing out the real causes of disease. Classification by districts and by streets shows a good deal to the intelligent investigator; but there can be little doubt that in the condition of the individual houses lies the real key to that of the district. The method adopted by Dr. Allen is an expansion of the well-known card index. For every house in the district there is a card giving certain particulars, such as the deaths and infectious diseases that have occurred in the house in question, the dates of inspections, of rebuilding, of draining, &c., and also a number referring to an envelope in which are deposited plans of drainage, copies of complaints, and other documents concerning the house. These envelopes are stored according to their consecutive numbering, and thus by reference to the cards, which are arranged according to streets, it is possible without waste of time to ascertain at once the sanitary history of any house in the district.

[1] Public Health, Sept.

